# Metabolic Modulators in Cardiovascular Disease Management

**DOI:** 10.3390/metabo16100703

**Published:** 2026-09-23

**Authors:** Syed Siraj A. Quadri

**Affiliations:** Debusk College of Osteopathic Medicine, Lincoln Memorial University, 9737 Cogdill Road, Knoxville, TN 37932, USA; syed.quadri@lmunet.edu

## 1. Introduction

Cardiovascular disease (CVD) remains the leading cause of morbidity and mortality worldwide, driven in large part by the complex interactions among metabolic dysfunction, inflammation, and maladaptive cellular remodeling. As our understanding of these mechanisms deepens, so too does the opportunity for targeted, metabolism-centered therapeutic strategies. This Special Issue, “Metabolic Modulators in Cardiovascular Disease Management”, was conceived with the intent to bring together cutting-edge research that bridges molecular metabolism with clinical cardiovascular outcomes.

Over the course of this Special Issue spanning August 2025 to May 2026, five articles were published. Each study addressed a distinct node in the metabolic–cardiovascular axis from intracellular quality control pathways and epigenetic regulation to novel pharmacological agents and regenerative cell therapies. As Guest Editor, it is both a privilege and a responsibility to close this collection with a synthesis of the major scientific insights this Special Issue offers.

## 2. Summary of Published Articles

### 2.1. SGLT2 Inhibitors: Multifaceted Therapeutic Agents in Cardiometabolic and Renal Diseases

*Checa-Ros A, Okojie O-J, D’Marco L. Metabolites 2025, 15(8), 536.*

Opening this Special Issue, this comprehensive review by Checa-Ros et al. provides an authoritative synthesis of the pleiotropic mechanisms underlying the cardiovascular and renal benefits of sodium-glucose cotransporter-2 (SGLT2) inhibitors. The authors extend the pharmacological narrative well beyond glycemic control, demonstrating that SGLT2 inhibitors exert meaningful effects through hemodynamic unloading, reduction in oxidative stress, attenuation of inflammation, and direct modulation of cardiac energy substrate utilization. Of particular note is the mechanistic discussion of SGLT2-inhibitor-driven ketogenesis and its role in providing an alternative, metabolically efficient fuel source for the failing heart—a concept that has reshaped our understanding of cardiometabolic pharmacotherapy. The clinical breadth of this review, encompassing heart failure with reduced and preserved ejection fraction as well as chronic kidney disease, establishes a foundation for the metabolic themes developed throughout the remainder of this Special Issue.

### 2.2. Targeting Metabolic Dysregulation in Obesity and Metabolic Syndrome: Emerging Role of N-Acetylcysteine

*Radomska-Leśniewska DM, Niderla-Bielińska J, Kujawa M, Jankowska-Steifer E. Metabolites 2025, 15(10), 645.*

This article introduces N-acetylcysteine (NAC) as a pharmacologically versatile agent in the management of metabolic syndrome and obesity-related cardiovascular risk. The authors detail how NAC replenishes intracellular glutathione reserves, thereby mitigating the oxidative stress that is central to the pathogenesis of insulin resistance, dyslipidemia, and endothelial dysfunction. The review further explores NAC’s anti-inflammatory properties, its capacity to modulate adipokine profiles, and its emerging role in mitigating nonalcoholic fatty liver disease—a key metabolic comorbidity in patients with cardiovascular disease. This work has attracted substantial community interest, garnering four citations and over 4,000 views following publication, reflecting the broad clinical relevance of repurposing an established antioxidant in the context of modern cardiometabolic disease management.

### 2.3. Cardiometabolic Therapies Shape Noncoding RNA Landscapes in Cardiovascular Fibrosis

*Floris E, Nutile F, Cozzolino C, Pontecorvi V, Bordin A, De Falco E, Picchio V, Chimenti I, Pagano F. Metabolites 2025, 15(10), 664.*

Floris et al. contributed an investigation into the epigenomic consequences of cardiometabolic pharmacotherapy, with a particular focus on how current therapeutic agents modulate the noncoding RNA (ncRNA) landscape in the context of cardiovascular fibrosis. The study demonstrates that drugs commonly used in cardiometabolic disease—including statins, SGLT2 inhibitors, and GLP-1 receptor agonists—exert previously underappreciated regulatory effects on the microRNAs, long noncoding RNAs, and circular RNAs implicated in fibroblast activation and extracellular matrix remodeling. This work opens a translational avenue: the identification of drug-modulated ncRNA signatures may serve as biomarkers of therapeutic efficacy and novel targets for antifibrotic intervention. The article broadens the molecular frame of reference within this Special Issue, underscoring that metabolic modulation extends to the epigenetic level.

### 2.4. Recent Advances in Pluripotent Stem Cell-Derived Cardiomyocytes for Regenerative Medicine

*Ibrahim FM, Atef A, Mostafa MM, Sayed MA. Metabolites 2025, 15(11), 735.*

This review by Ibrahim et al. provides an appraisal of the state of the art in pluripotent stem cell-derived cardiomyocyte (PSC-CM) technology and its application in cardiac regenerative medicine. The authors address key challenges in the field—including the metabolic immaturity of PSC-CMs relative to adult ventricular cardiomyocytes—and present emerging strategies to overcome this limitation through metabolic substrate switching, including the promotion of fatty acid oxidation over glycolysis to achieve a more physiologically relevant metabolic phenotype. The convergence of cellular metabolism and regenerative potential highlighted in this article reinforces a central theme of this Special Issue: metabolic reprogramming is not merely a consequence of cardiovascular disease but a lever through which cardiac function can be restored. The authors also reviewed progress in disease modeling, drug screening platforms, and clinical translation pathways.

### 2.5. Regulation of Mitophagy by Low-Intensity Pulsed Ultrasound Attenuates Endothelial Dysfunction

*Shi Y, Zhao B, Wei Y, Lu D, Liu H, Chu Y. Metabolites 2026, 16(5), 329.*

The most recent contribution to this Special Issue offers an innovative mechanistic study linking low-intensity pulsed ultrasound (LIPUS)—a physical therapeutic modality—to the regulation of mitophagy and its downstream effects on endothelial dysfunction. Shi et al. found that LIPUS stimulation activates selective autophagic clearance of damaged mitochondria (mitophagy) in endothelial cells, thereby reducing reactive oxygen species generation, preserving nitric oxide bioavailability, and attenuating the inflammatory phenotype associated with cardiovascular risk. The authors introduce a nonpharmacological dimension to the metabolic modulation theme and highlight mitochondrial quality control as a targetable node in endothelial homeostasis. The findings have implications for the development of adjunctive, noninvasive interventions in patients with atherosclerosis, hypertension, and diabetes-associated endothelial injury.

## 3. Integrative Perspectives and Emerging Themes

Viewed collectively, the five articles in this Special Issue provide a rich and multidimensional narrative on the role of metabolic modulators in cardiovascular health. Several cross-cutting themes emerge.

**First, mitochondrial biology occupies a central position** in multiple contributions. The LIPUS mitophagy study (Shi et al.) and the PSC-CM metabolic maturation discussion (Ibrahim et al.) converge on the premise that mitochondrial health—whether restored via autophagic clearance or achieved through metabolic substrate optimization—is indispensable to cardiovascular cellular function. SGLT2-inhibitor-mediated ketogenesis (Checa-Ros et al.) similarly reflects a shift toward mitochondria-favorable fuel metabolism in the stressed heart.

**Second, oxidative stress and redox signaling** appear as recurring pathological drivers. The mechanistic rationale for NAC in metabolic syndrome (Radomska-Leśniewska et al.) and the mitophagy-mediated preservation of nitric oxide in endothelial cells (Shi et al.) underscore the therapeutic imperative of restoring redox balance in the cardiovascular system.

**Third, epigenomic regulation** represents a frontier that this Special Issue has helped define. The noncoding RNA landscape study (Floris et al.) signals an important evolution in how we conceptualize drug action—beyond receptor pharmacology toward gene regulatory networks—and positions ncRNA profiling as a promising tool for personalized cardiovascular medicine.

**Fourth, this Special Issue bridges basic science and translational application**. From bench-level mechanistic investigations to clinical pharmacology reviews and regenerative cell therapy, the presented research reflects the full continuum from discovery to bedside application—a hallmark of impactful biomedical science.

## 4. Conclusions

This Special Issue, “Metabolic Modulators in Cardiovascular Disease Management”, has highlighted the many ways in which cellular metabolism intersects with cardiovascular pathophysiology and therapeutics. The contributions gathered here reinforce the message that the heart and vasculature are fundamentally metabolic organs—organs whose integrity is shaped not only by hemodynamics and electrical activity but also by the energy substrates they utilize, the oxidative environment they inhabit, and the epigenomic programs that govern their responses to stress.

Looking ahead, several research priorities are indicated by studies assembled here. The integration of ncRNA biomarker profiling into clinical cardiometabolic monitoring warrants further prospective evaluation. The metabolic maturation of PSC-CMs remains an unsolved challenge that, once addressed, could revolutionize cardiac repair. The application of physical modalities such as LIPUS to regulate mitophagy and endothelial function deserves expansion into preclinical and early-phase clinical studies. Additionally, the polypharmacology of established agents such as SGLT2 inhibitors and NAC continues to yield mechanistic discoveries that may guide rational combination therapy.

As Guest Editor, I am deeply grateful to all contributing authors for their scholarly dedication and to the reviewers whose rigorous evaluations ensured the scientific quality of each manuscript. I extend my sincere appreciation to the editorial team at *Metabolites* for their professionalism and support throughout the publication process. I hope that this collection will serve as a valuable resource for researchers, clinicians, and students working at the intersection of metabolism and cardiovascular medicine and that it will inspire the next generation of discoveries in this vital field.

